# Transthyretin Aggregation Pathway toward the Formation of Distinct Cytotoxic Oligomers

**DOI:** 10.1038/s41598-018-37230-1

**Published:** 2019-01-10

**Authors:** Anvesh K. R. Dasari, Robert M. Hughes, Sungsool Wi, Ivan Hung, Zhehong Gan, Jeffrey W. Kelly, Kwang Hun Lim

**Affiliations:** 1 Department of Chemistry, East Carolina University, Greenville, NC 27858 USA; 2 Center of Interdisciplinary Magnetic Resonance (CIMAR), National High Magnetic Field Laboratory (NHMFL), 1800 East, Paul Dirac Dr., Tallahassee, FL 32310 USA; 3 Department of Molecular and Experimental Medicine, the Skaggs Institute for Chemical Biology, The Scripps Research Institute, La Jolla, CA 92037 USA

## Abstract

Characterization of small oligomers formed at an early stage of amyloid formation is critical to understanding molecular mechanism of pathogenic aggregation process. Here we identified and characterized cytotoxic oligomeric intermediates populated during transthyretin (TTR) aggregation process. Under the amyloid-forming conditions, TTR initially forms a dimer through interactions between outer strands. The dimers are then associated to form a hexamer with a spherical shape, which serves as a building block to self-assemble into cytotoxic oligomers. Notably, wild-type (WT) TTR tends to form linear oligomers, while a TTR variant (G53A) prefers forming annular oligomers with pore-like structures. Structural analyses of the amyloidogenic intermediates using circular dichroism (CD) and solid-state NMR reveal that the dimer and oligomers have a significant degree of native-like β-sheet structures (35–38%), but with more disordered regions (~60%) than those of native TTR. The TTR variant oligomers are also less structured than WT oligomers. The partially folded nature of the oligomeric intermediates might be a common structural property of cytotoxic oligomers. The higher flexibility of the dimer and oligomers may also compensate for the entropic loss due to the oligomerization of the monomers.

## Introduction

Protein misfolding and amyloid formation is implicated in various debilitating human diseases including Alzheimer’s diseases and amyloidoses^1–3^. The protein aggregation process involves conformational changes from native polypeptides to aggregation-prone intermediates that self-assemble into β-structured amyloid. Recent studies demonstrated that amyloid formation of even a single protein can proceed via multiple misfolding pathways through the formation of multiple misfolding intermediates, leading to distinct amyloid with different molecular structures^4–10^. Identification and characterization of various cytotoxic species populated during the complicated aggregation process would be of great importance in developing therapeutic strategies for the protein misfolding disorders.

Increasing evidence suggested that small oligomers formed at an early stage of amyloid formation are real cytotoxic species^11–19^. Characterization of oligomeric intermediate states is, therefore, critical to understanding the molecular mechanism of pathogenic oligomerization process^20–22^. Investigation of the small oligomers has, however, been of great challenge due to their transient, heterogeneous nature^12,13,23^. In this study, we report a detailed molecular mechanism of transthyretin (TTR) oligomerization process through characterization of small intermediate states of WT and a mutant form of TTR (G53A).

TTR is a 55 kDa homo-tetrameric protein that binds and transports thyroxine and holo-retinol binding protein in the cerebrospinal fluid and plasma^24^. TTR misfolding and amyloid formation is associated with numerous amyloidoses including senile systemic amyloidosis, familial amyloidotic polyneuropathy, and familial amyloid cardiomyopathy^25–27^. Previous studies showed that native tetrameric TTR is dissociated to monomers that undergo a local conformational transition to amyloidogenic monomers^28–31^. The aggregation-prone monomers effectively self-assemble into insoluble amyloid via downhill mechanism^32^, and thus isolation of oligomeric intermediate states for biochemical characterization has been a daunting task. In this report, we isolated small oligomeric species populated during TTR oligomerization process and investigated structural features of the cytotoxic oligomeric species of WT and a mutant form of TTR (G53A). Our combined analyses of the oligomeric species provided valuable insights into aggregation pathways to the formation of two distinct cytotoxic oligomers and into the structural features of the oligomeric species.

## Results and Discussion

**Figure 1:**
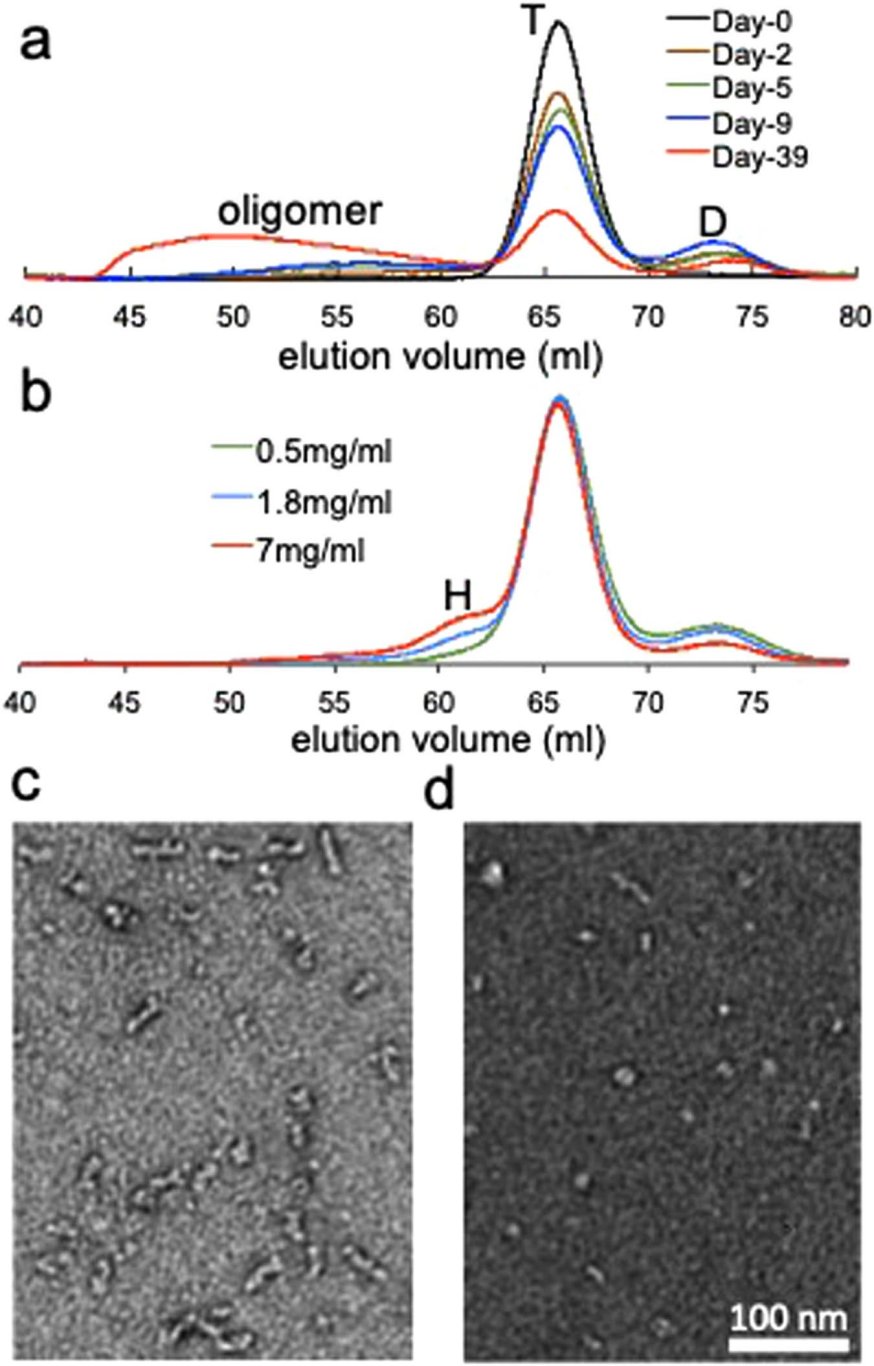
(**a**) SEC analyses of TTR samples (2 mg/ml) aged at different incubation times at 4 °C using Superdex 200 size exclusion column (GE Healthcare). The TTR samples were incubated in 20 mM sodium acetate buffer (pH 4.4) and 300 μL of the sample was injected to the column after the pH was adjusted to 7. T and D denote tetramer and dimer, respectively. The protein eluting around 73 ml was determined to be a dimer by mass spectrometry (Fig. S1). (**b**) SEC analyses of TTR samples (uncross-linked) at different concentrations (total monomer concentration) at 4 °C. A mixture of dimeric and native TTR purified by SEC was concentrated to the different concentrations and subjected to the SEC column without further incubation. (**c**) TEM image of the TTR oligomer eluting at 53 ml in Fig. 1a. (**d**) TEM image of the TTR oligomer eluting at 61 ml. The elution volumes of the hexamer (H), tetramer (T), and dimer (D) are slightly higher than those of the cross-linked TTR shown in Fig. S2.

In order to detect small oligomeric species that may form at an early stage of aggregation, we incubated wild-type (WT) TTR under the amyloidogenic pH of 4.4 at low temperature (4 °C). After various incubation times, the pH of the protein sample was increased to the neutral pH to slow down the aggregation kinetics, and the aged TTR sample was analyzed using size-exclusion chromatography (SEC, Fig. 1a). It was previously shown that WT tetrameric TTR dissociates to monomers at the amyloidogenic pH of 4.4. Ultracentrifugation experiments showed that approximately 25% of the tetramers are observed at a protein concentration of 0.2 mg/ml^28^. Thus a substantial amount of native tetramer may exist in the TTR sample (2 mg/ml), which elutes at about 66 ml in Fig. 1a. After longer incubations, the amount of native tetramers gradually decreases, while bigger oligomers were formed during the aggregation process (Fig. 1a). It is also interesting to note that TTR forms a dimer eluting at around 73 ml at an early stage of the aggregation. The dimers appear to self-assemble into bigger oligomers with elution volumes of less than 63 ml (Fig. 1a).

In order to more clearly investigate the early stage of oligomer formation, TTR dimers that were isolated in Fig. 1a were concentrated and analyzed by SEC (Fig. S2). At a higher dimer concentration (2 mg/ml, monomer concentration), the dimers are oligomerized to hexamers and bigger oligomers (red in Fig. S2). The formation of hexamers was more clearly observed at a lower protein concentration (Fig. 1b). TTR samples aged at a short period of time that contain only native tetramers and dimers were concentrated at pH 7.4 to investigate the formation of oligomers from the dimers (Fig. 1b). At higher concentrations, a small oligomer was observed at an elution volume of ~61 ml. Bigger oligomers with an elution volume of <~57 ml were also detected at a higher concentration of 7 mg/ml. The amount of dimers gradually decreases at higher protein concentrations, while tetramer concentration remains unchanged, suggesting that the dimers directly form the oligomers. The elution volume of the smaller oligomers was compared to those of the cross-linked TTR tetramers and octamers (Fig. S2). The small oligomers elute between the tetramers and octamers, indicating that the small oligomer is a hexameric TTR.

In the SEC analyses of the small oligomers described above, the TTR samples were injected after the pH was adjusted from the amyloidogenic pH (4.4) to the neutral pH. The morphology of the oligomers was investigated with transmission electron microscopy (TEM) to ensure that the change in pH does not induce oligomerization or disassemble the oligomers (Fig. S3). The TEM image of the TTR samples incubated at pH 4.4 exhibits heterogeneous mixtures of small oligomers with spherical shape and bigger oligomers. It is also notable that the bigger aggregates consist of the small spherical oligomers, suggesting that the spherical oligomers self-assemble into bigger aggregates. In addition, the morphology of the oligomers were not affected by the pH change (Fig. S3), indicating that the change in pH dose not induce structural changes of the oligomers.

The SEC analyses and TEM images of the TTR oligomers suggest that the amyloidogenic TTR dimers form a hexamer that may correspond to the spherical small oligomers in the TEM image shown in Fig. S3. The morphology of the hexamers eluting at ~61 ml and bigger oligomers eluting at ~53 ml were examined using TEM (Fig. 1c,d). The TTR aggregates with an eluting volume of 53 ml appear to consist of small oligomers with a spherical shape (Fig. 1c). The spherical small oligomers were observed for the hexamers eluting at about 61 ml (Fig. 1d), supporting that TTR hexamers are associated to form bigger aggregates.

**Figure 2:**
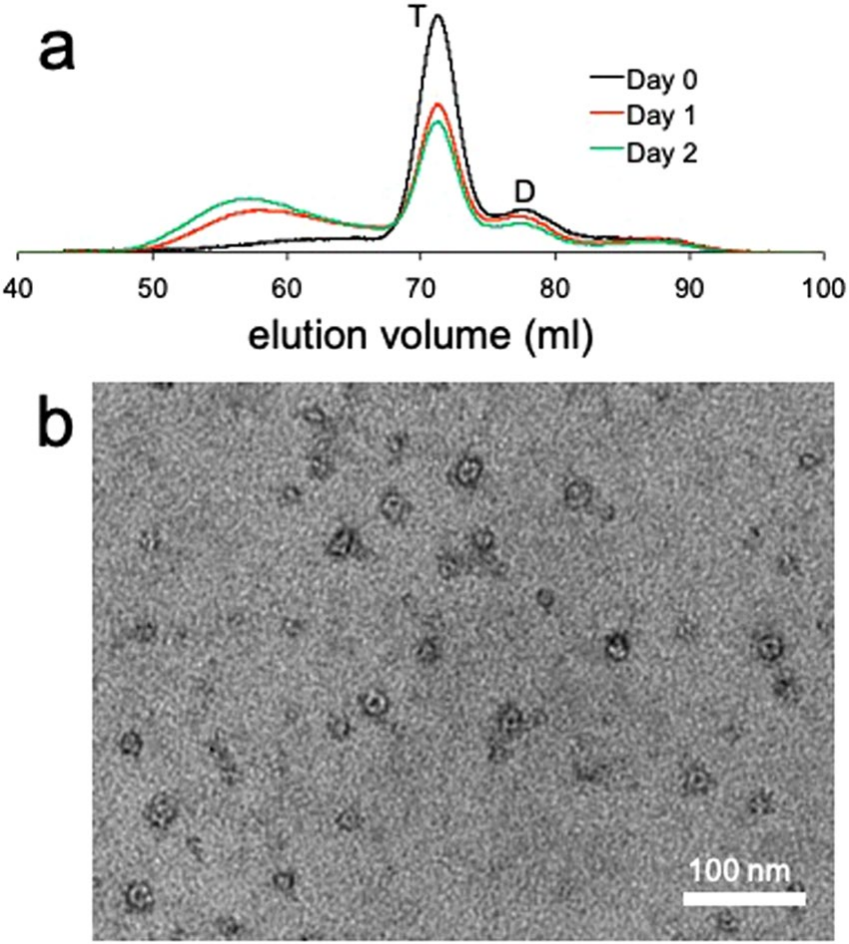
(**a**) SEC analyses of the G53A TTR (1 mg/ml) aged at different incubation times at 4 °C using Superdex 200 size exclusion column. The TTR variant was incubated in 20 mM PBS buffer (pH 7.4) and 300 μL of the sample was injected to the column. T and D denote tetramer and dimer, respectively. (**b**) TEM image of the G53A TTR oligomers purified by SEC.

The misfolding and oligomer formation of wild-type (WT) TTR was induced at mildly acidic conditions. Although the misfolding studies under the non-physiological conditions have provided valuable insights into the TTR misfolding pathways^33^, it is unclear whether TTR aggregation proceeds by the similar oligomerization mechanism at physiologically relevant conditions. Thus a TTR variant (G53A) associated with oculoleptomeningeal amyloidosis was used to explore misfolding process under the physiological pH. Notably, the pathogenic TTR variant readily forms amyloid at the physiological pH. The aggregation process was examined at the low temperature (4 °C) to probe oligomeric species using SEC (Fig. 2a). The SEC analyses at different incubation times show that the TTR variant also forms a dimer, which then self-assembles into oligomers. Interestingly, the G53A TTR variant forms mainly annular oligomers with diameters of 15–25 nm (Fig. 2b). The annular oligomers with pore-like structures appear to consist of small spherical species, as was observed in WT TTR oligomers (Fig. 1c). These combined results suggest that native tetrameric TTR dissociates into monomers that form dimers first. The dimers are then associated to form hexamers, which serves as a building block to self-assemble into linear oligomers (WT TTR) and annular oligomers with pore-like structures (G53A).

**Figure 3:**
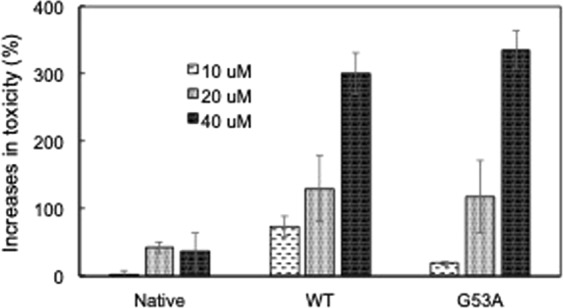
Increases in cytotoxicity of native TTR, and WT and G53A oligomers assessed using SH-SY5Y cells with LIVE/DEAD cell viability assay (Molecular Devices, CA). The mammalian cells were treated with native WT TTR and the oligomers at different concentrations (monomer concentration) for 48 hours, and the number of live and dead cells was analyzed. The oligomers were isolated from TTR samples (1 mg/ml) incubated at 4 °C for one week (Fig. S4).

There is an increasing body of evidence that suggests small oligomeric intermediates are real cytotoxic species. Cytotoxicity of the oligomers formed by WT and G53A TTR isolated by SEC in Fig. S4a,b, respectively, were assessed on SH-SY5Y neuroblastoma cells using cell viability assays (Fig. 3). Cytotoxicity of the oligomers gradually increases at higher oligomer concentrations, suggesting that the oligomeric species have cytotoxic activities, consistent with previous observations^16,34^. The G53A TTR oligomers appear to have similar cytotoxic activities to those of WT TTR oligomers. The TEM image of the TTR oligomers tested for the cell viability show that WT oligomeric species are heterogeneous mixtures of small oligomers (Fig. S5a). Previous studies showed that small TTR oligomers (<100 kDa) are more toxic than bigger aggregates^16,34^, suggesting that the small spherical oligomers (hexamer) may be real toxic species. On the other hand, G53A TTR oligomers contain annular oligomers with central cavities (Fig. S5b). In particular, the G53A oligomers consisting of six to seven small hexameric spherical oligomers have effective cytotoxic activity considering low particle concentrations (less than 1 μM for a monomer concentration of 40 μM in Fig. 3). More importantly, the TTR variant forms cytotoxic oligomers much faster even under the physiological pH than WT TTR at mildly acidic condition (Figs 1a and 2a), which correlates well with much earlier onset of oculoleptomeningeal amyloidosis associated with G53A mutation.

**Figure 4:**
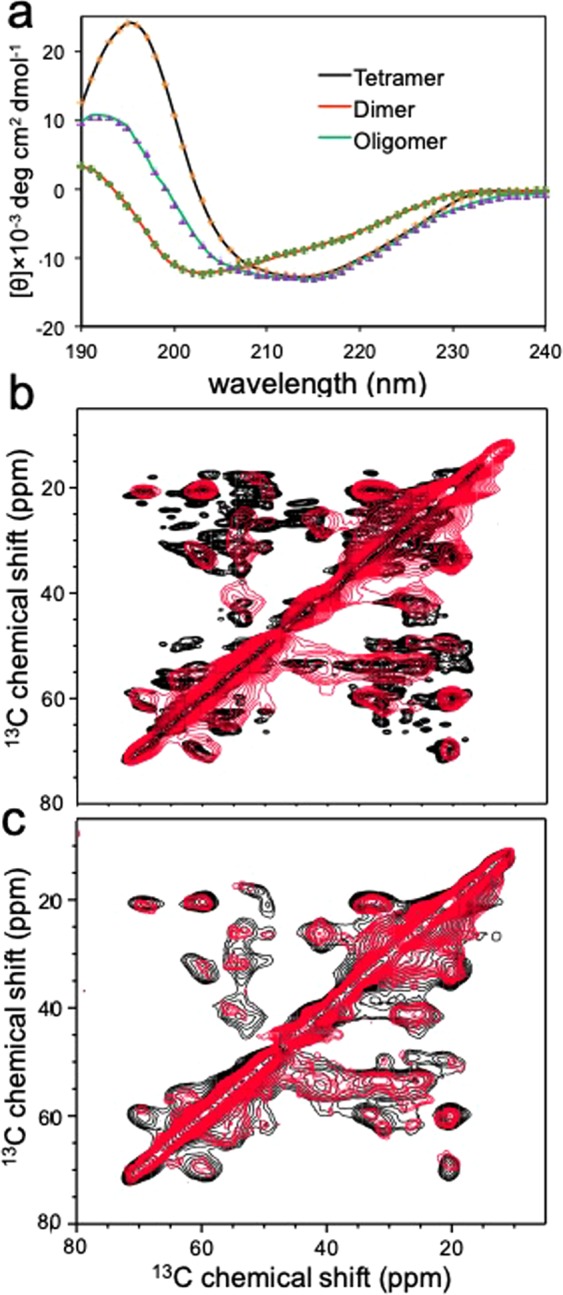
(**a**) Experimental CD spectra of tetrameric WT TTR (black), dimer (red), and oligomer (green) eluting at 61 mL in Fig. 1, along with reconstructed dotted spectra using the software DichroWeb. The protein concentration of 0.2 mg/ml (monomer concentration) was used for the protein samples. (**b**) Overlaid two-dimensional (2D) ^13^C-^13^C correlation solid-state NMR spectra of WT (native) and WT oligomer (red) obtained with COmbined R2n(v)-Driven (CORD)^58^ recoupling scheme. (**c**) Overlaid 2D spectra of WT (black) and G53A (red) oligomers. The CORD mixing time was 115 ms.

Structural features of the WT dimers and oligomers observed in SEC (Fig. 1) were investigated using circular dichroism (CD) spectroscopy (Fig. 4a). The CD spectra of the dimer and native tetramer exhibit a marked difference, particularly in low wavelength regions. The positive maximum and negative minimum at 197 nm and 214 nm, respectively, in the native CD spectrum correspond to a typical β-sheet conformation. On the contrary, the CD signal below 200 nm is negative in the dimer CD spectrum, suggesting that the dimer becomes more disordered. The small oligomer exhibits an intermediate CD spectrum between the dimer and native tetramer, particularly in lower wavelength regions (<205 nm). The stronger signals at 205–220 nm than those of the dimer suggest that the oligomers have more β-sheet and α-helical content. However, the weaker signals at the low wavelength regions (<205 nm) compared with those of native tetramer indicate that the oligomers are substantially more disordered than the native tetramer. The structural features of the dimer and oligomer were further analyzed using the software DichroWeb^35,36^ (Table 1).

**Table 1:** Secondary structural analyses of native TTR, dimer, and oligomer using the software DichroWeb.

	α-helix (%)	β-sheet (%)	disordered (%)	RMSD
Native (pdb: 1F41))	5.5	48	46.5	—
WT Native	7	42	51	0.012
Dimer	3	34.5	62.5	0.023
Oligomer	6	38	57	0.022

The secondary structural analyses of the CD spectra revealed that the dimer contains a considerable amount of β-sheet structure (35%, Table 1). The amyloidogenic dimers also appear substantially disordered with less α-helical content (3%) compared with tetrameric native TTR (7%). The small oligomeric species become slightly more structured than the dimers, but still contain extensive disordered regions with some helical content, suggesting that the β-sheet structures characteristics of amyloid fibrils are not fully developed in the oligomeric intermediate states.

The structural features of the WT oligomers were compared to those of native TTR using solid-state NMR (Fig. 4b). In the cross-polarization (CP) based solid-state NMR spectra, the strong ^13^C-^13^C correlation cross-peaks originate mainly from residues in rigid, structured amyloid core regions^37–39^. It was also shown that NMR peaks are usually not observed from flexible and/or disordered regions due to severe line-broadening and/or inefficient cross-polarization caused by motional averaging of dipolar interactions^37–39^. Thus, the strong NMR cross-peaks from WT oligomers (red) shown in Fig. 4b suggests that the oligomers contain rigid, structured regions. It is also notable that the NMR peaks from the oligomers are overlapped well with those of native state TTR (black), indicating that the oligomeric TTR contains native-like β-structures. The substantial β-sheet conformations in the WT oligomers are further evidenced by the chemical shift values of the sidechains (Fig. S6). However, NMR resonances from the oligomers are substantially broader, and many of the cross-peaks in native TTR are not observed in the oligomer spectrum. These NMR results indicate that the WT oligomeric states are substantially more disordered than the native state, which is in good agreement with the CD spectra (Fig. 4a).

The structural feature of the oligomeric state was also compared to that of the final product, amyloid (Fig. S7). The 2D solid-state NMR spectra for the two states are also overlapped well, suggesting that the two states have similar structural features. Our previous solid-state NMR studies showed TTR amyloid state contains extensive native β-sheet conformations^40^, suggesting that the native-like β-sheet structures are maintained in the oligomeric and amyloid states of TTR. These results also support that the hexameric oligomers serve as the building block that self-assembles into the bigger aggregates. It is, however, notable that some of the resonances in the amyloid state are not observed in the oligomer spectrum, indicating that the β-structure is not fully developed in the oligomeric state consistent with the CD spectrum (Fig. 4a).

The 2D solid-state NMR spectrum was also acquired for G53A oligomer and compared to that of WT oligomer (Fig. 4c). The similar NMR spectra suggest that the two oligomeric states possess similar structural features. However, NMR cross-peaks for the G53A oligomer are much weaker in intensity than those of WT oligomer, implying that G53A oligomer is more disordered than WT oligomer. The structural differences may result in distinct morphologies observed in TEM.

**Figure 5:**
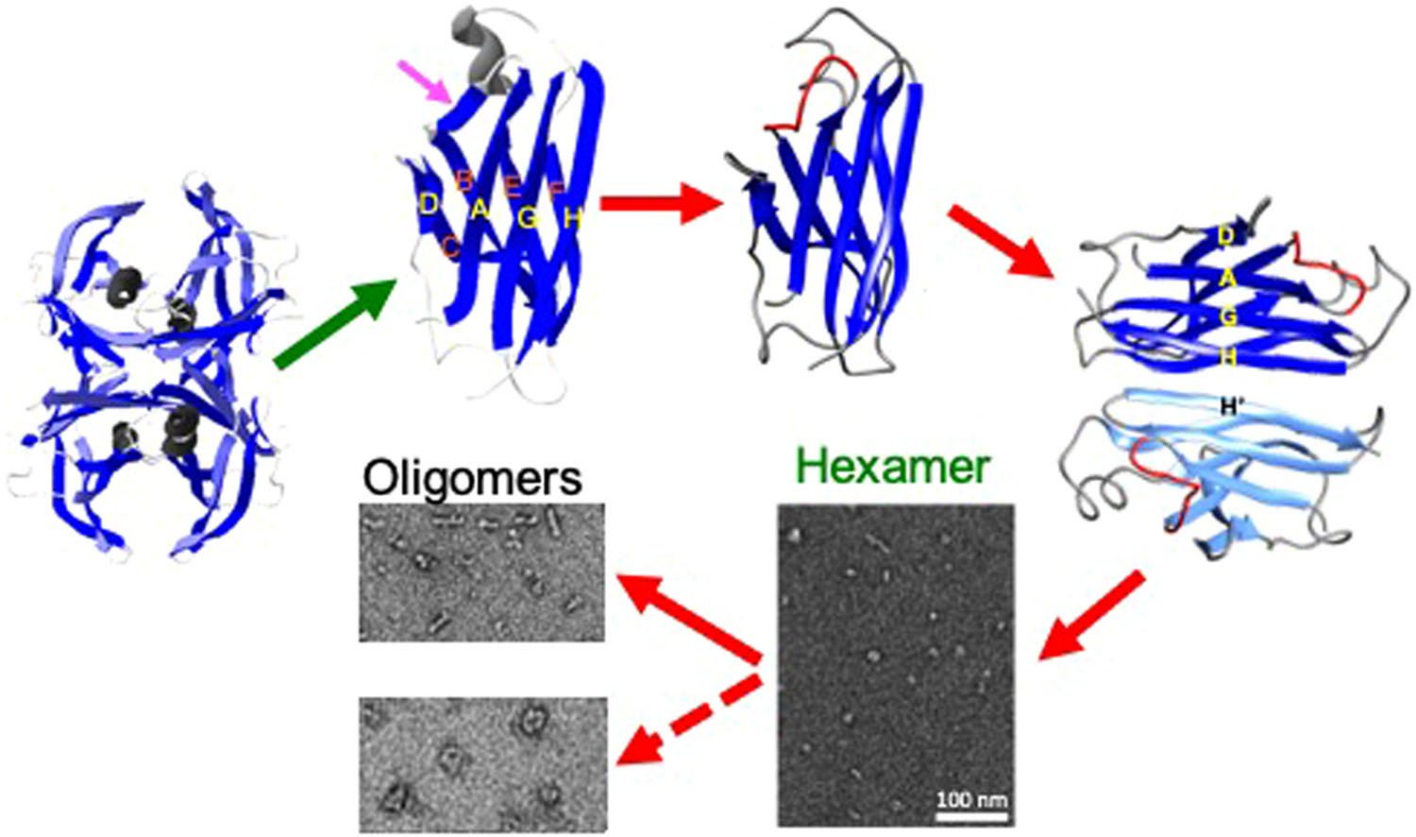
Proposed molecular mechanism of TTR aggregation on the basis of our biochemical analyses and previous experimental results. Under amyloidogenic conditions, tetrameric native TTR is dissociated to monomers, which undergo local conformational changes in AB loop region (pink arrow)^31^. The amyloidogenic monomers form a dimer through H-H′ interactions. The dimers are then associated to form spherical hexamers, which self-assemble into linear (WT TTR) or annular oligomers (G53A). The structural models for the native tetramer, monomers, and dimers were drawn using MOLMOL^60^ with pdb code (1F41)^61^.

Although the nature of cytotoxic species is still under intense debate^13,15,23,41^, there is a growing body of evidence that suggested small oligomeric species are real cytotoxic agents. The cytotoxic oligomers are only transiently populated and highly inhomogeneous, and thus isolation and characterization of the cytotoxic species are of great challenge. In this study, we identified cytotoxic oligomeric intermediates formed by natively folded protein (TTR). Our combined analyses of the intermediate states formed at the early stage of aggregation revealed molecular details of aggregation pathways toward the formation of distinct cytotoxic TTR oligomers. TTR is a natively β-structured protein that contains two four-β-stranded anti-parallel sheets (CBEF and DAGH) arranged into a β-sandwich (Fig. 5)^42–44^. Our previous solid-state NMR studies revealed that the native-like CBEF and AGH β-structure is retained, while AB loop and helical regions become more disordered during TTR aggregation process^31,40,45^, suggesting that outer strands (C, A, F, and H) are available for intermolecular association. Previous mechanistic studies of TTR aggregation revealed that intermolecular interactions between strands H play a key role in TTR amyloid formation^46,47^.

On the basis of the current and previous studies, we propose that amyloidogenic monomers dissociated from native tetramers form a dimeric intermediate state through H-H′ interactions, and the dimers are associated to form hexamers (Fig. 5). The spherical hexamers appear to serve as a building block that self-assembles into various bigger oligomeric species. Interestingly, WT TTR tends to form linear oligomers, while a TTR variant (G53A) prefers forming annular oligomers with pore-like structures. The annular oligomers with central cavities have been observed for other pathological aggregation-prone proteins including Aβ peptides and α-synuclein^11,48,49^. It was proposed that the pore-forming oligomers act like a non-selective ion channel that disrupts the membrane potential^49,50^. In addition to the pore-forming oligomers, other types of oligomers without pore-like structures such as WT TTR oligomers were also shown to exhibit cytotoxic activities^12,51^, suggesting that shape of the oligomers might not be a crucial factor for the toxicities. Our combined structural analyses of the two cytotoxic oligomers using CD and solid-state NMR showed that the oligomeric states contain considerable β-sheet structures, but with extensive disordered regions. The intermediate content of β-sheet structures with a high degree of disorderness might be a more important feature of the cytotoxic oligomers than the shape of the oligomers^12,13,23,52,53^. The high flexibility of the oligomeric species may also help compensate for entropic loss during oligomerization process.

## Conclusion

We report two distinct cytotoxic oligomers formed at the mildly acidic (WT TTR) and physiological pH (G53A variant). The TTR amyloidoses is characterized by extreme variations of the disease phenotype^54^. For example, aggregation of wild-type (WT) TTR affects primarily the heart and lung. Various single-point mutations including V30M and L55P cause exclusively neurological disorders (polyneuropathy), and rare mutations such as G53A and A25T are associated with oculoleptomeningeal amyloidosis. Previous biophysical studies showed that monomeric TTR dissociated from tetrameric TTR can adopt various partially unfolded conformational states in equilibrium^55,56^. Thus, the pathogenic single point mutations may shift the equilibrium to a distinct conformational state, leading to a different aggregation pathway, which is demonstrated for WT and G53A TTR in this report. Further analyses of other mutant forms of TTR will provide additional insights into distinct misfolding pathways of TTR variants that may cause tissue-selective TTR amyloidosis.

## Materials and Methods

### Transthyretin (TTR) expression and purification

Recombinant wild-type (WT) and a mutant form of TTR were expressed and purified from an *Escherichia coli* cells transformed from the pMMHa plasmid as described previously^57^. Briefly, a starter culture of 5 mL of Luria-Bertani (LB) medium was inoculated with a single colony and grown until OD_600_ reaches 0.6. The starter culture was then transferred to 50 mL LB medium. At OD_600_ of 0.6, the 50 ml culture was transferred to a 500 mL of LB medium and grown until OD_600_ reaches 0.6. The cultures were induced with addition of IPTG to a final concentration of 1 mM, and the protein was overexpressed at 25 °C overnight. Cells were harvested after OD_600_ reaches around 1.6 and the pellet was stored at −80 °C. Cells were resuspended in the lysis buffer (20 mM Tris, 150 mM NaCl) and disrupted by sonication. The cell lysates were then centrifuged to collect the super and the supernatant was precipitated with 50% ammonium sulfate. After centrifugation, the supernatant was saved and dialyzed against 20 mM Tris buffer (with 1 mM EDTA and 1 mM PMSF). Anion exchange chromatography (HiTrap Q HP 10 mL, GE Healthcare) and size exclusion chromatography (HiLoad 16/60 Superdex 200 column, GE Healthcare) were used for further protein purification. All the cultures were supplemented with carbenicillin (100 mg/mL) and were shook at 250 rpm at 37 °C.

### WT TTR oligomer preparation for the cytotoxicity test

WT TTR exists as a natively folded tetrameric state at the neutral pH. Thus, the purified WT TTR (10 mg/ml in 10 mM phosphate buffer, pH 7.4) was diluted with 20 mM sodium acetate buffer (pH 4.2) to a final concentration of 1 mg/mL at pH 4.4 to induce misfolding and aggregation under the mildly acidic condition. The WT TTR at pH 4.4 was incubated for 7 days at 4 °C. The pH of the protein sample was then increased to 7.4 by addition of NaOH at 4 °C to slow down aggregation kinetics. The resulting TTR sample at the neutral pH was concentrated by ten times using Amicon® stirred cells and injected into size-exclusion gel filtration column (SEC, HiLoad 16/60 Superdex 200, GE Healthcare). The oligomer fractions eluting between 55–60 ml in Fig. S4a were collected used for the toxicity test.

### G53A TTR oligomer preparation for the cytotoxicity test

The G53ATTR variant is purified as a natively folded tetrameric state at the neutral pH. Unlike WT TTR, the TTR mutant can form amyloid at the physiological pH, and thus the purified G53A TTR (1 mg/mL in 10 mM phosphate buffer) was incubated for 7 days at 4 °C at the physiological pH. The G53A TTR samples incubated for 7 days were then subject to the SEC Superdex 200 column to collect the TTR oligomers eluting from 55 to 60 ml in Fig. S4b.

### Cross-linking of TTR

Glutaraldehyde (Sigma) was used to initiate cross-linking reactions of TTR. Fifty microliters of the cross-linking agent (25% solution) were mixed with 500 μL of TTR (0.5 mg/ml) at 25 °C. After 4 minutes of incubation, the reaction was quenched by addition of 50 μL of 7% solution of NaBH_4_ (wt/vol in 0.1 M NaOH).

### Cell culture

Human neuroblastoma SH-SY5Y (ATCC^®^ CRL-2266^™^) cells were grown in complete growth medium (1:1 mixture of DMEM/F12 medium with 10% FBS and 1% Pen-Strep) at 37 °C in 5% CO_2_. The medium was renewed every 4–5 days.

### Cell viability assay

Cells were plated (96-well clear bottom black plates) at a density of 10,000–15,000 cells/well one day before the treatment to let the cell attach to the surface. All TTR samples were buffer exchanged with complete growth medium just before the treatment and filter sterilized. Cell medium from wells was replaced with TTR solutions and incubated at 37 °C in 5% CO_2_ for 2 days. All TTR solutions were tested in triplicate at three dilutions. Blanks were prepared by adding the complete growth medium to the wells with no cells.

For the Live/Dead assay, 100 µL of calcein AM/EthD-III solution (3 μM each in PBS) was added to each well and incubated the plates at room temperature for 45 min. Live/dead cell ratio was calculated by measuring fluorescence at Ex_495_/Em_530_ for live cells and at Ex_530_/Em_645_ for dead cells. The relative live/dead ratios were normalized to fluorescence ratios from the vehicle (complete growth medium) only wells.

### SEC

TTR samples were filtered through 0.22 μM filter and injected into HiLoad 16/60 Superdex 200 column and eluted with 5 mM or 10 mM phosphate buffer at room temperature or 4 °C.

### TEM

After the TTR samples (1–2 mg/ml) were diluted by 200 times in 5 mM phosphate buffer at 4 °C, 5 µL droplet was placed on formvar/carbon coated copper 400 mesh grids for 30 s and excess solution was blotted off with a filter paper. Grids were washed with 10 µL 1% uranyl acetate solution and stained with another 10 µL 1% uranyl acetate solution for 30 s. The excess staining solution was blotted off with a filter paper and the grids were air-dried. TEM images were acquired using Philips CM12 Transmission Electron Microscope at 80 kV.

### CD spectroscopy

CD spectra were recorded using 1 mm pathlength cuvette and JASCO J-815 CD spectrometer at room temperature using the protein concentrations of 0.15–0.25 mg/mL (pH 7.4). Thirty scans were accumulated for each protein samples and the CD signals from the buffer were subtracted. Secondary structural content of the proteins was calculated using the software DichroWeb.

### Solid-state NMR

Solid-state NMR spectra were recorded using Bruker 600 MHz spectrometer equipped with a 1.3 mm MAS probe. Only small amount of oligomer samples (1–2 mg) could be prepared from a large protein stock (10–20 mg), and thus 1.3 mm MAS probe was used for solid-state NMR experiments. Cross-polarization (CP) based two-dimensional ^13^C-^13^C solid-state NMR spectra were acquired using a COmbined R2n(v)-Driven (CORD) recoupling mixing scheme^58^ with ^1^H radio-frequency (rf) field strengths of 30 and 15 kHz for R2_1_^*v*^ and R2_2_^*v*^ symmetry sequences, respectively. A linear amplitude ramp on the ^1^H channel was used for the ^1^H/^13^C cross-polarization with a contact time of 1 ms.

The 90° pulse-lengths for ^1^H and ^13^C were 2.1 and 5 μs, respectively, and the SPINAL-64 (small phase incremental alternation with 64 steps) decoupling scheme^59^ was employed with an rf field strength of 90 kHz. For the 2D correlation NMR spectra, complex data points of 1024 × 290 were collected with an acquisition delay of 3 sec.

For the solid-state NMR experiments, oligomeric TTR was precipitated using 10–20% (NH_4_)_2_SO_4_ and the precipitates were washed with deionized water to remove soluble native TTR. The native state TTR begins to precipitate at much higher concentrations of NH_4_SO_4_ (>50%). In addition, the NMR cross-peaks of the native TTR were not observed in the NMR spectrum of the oligomeric states (Figs 4b,c), suggesting that only oligomeric states of TTR are present in the NMR sample. The effect of the (NH_4_)_2_SO_4_ on the oligomeric state was examined with TEM (Fig. S8) and the morphology of the oligomeric state was not affected by the salt.

## Supplementary information

Supporting Info

## Data Availability

All data generated or analyzed during this study are included in this published article (and its Supplementary Information files).
