# Supplementary material for: Transthyretin Aggregation Pathway toward the Formation of Distinct Cytotoxic Oligomers
Source: Sci Rep. 2019 Jan 10;9:33. doi: 10.1038/s41598-018-37230-1 (PMC6328637; doi:10.1038/s41598-018-37230-1)
Supplement: Supplementary file 1 — Supporting Info [file 41598_2018_37230_MOESM1_ESM.pdf]

Supporting Information

Transthyretin Aggregation Pathway toward the Formation of Distinct Cytotoxic Oligomers

Anvesh K. R. Dasari[a], Robert M. Hughes[a], Sungsool Wi[b], Ivan Hung[b], Zhehong Gan[b], Jeffrey W. Kelly[c], and Kwang Hun Lim*,[a]

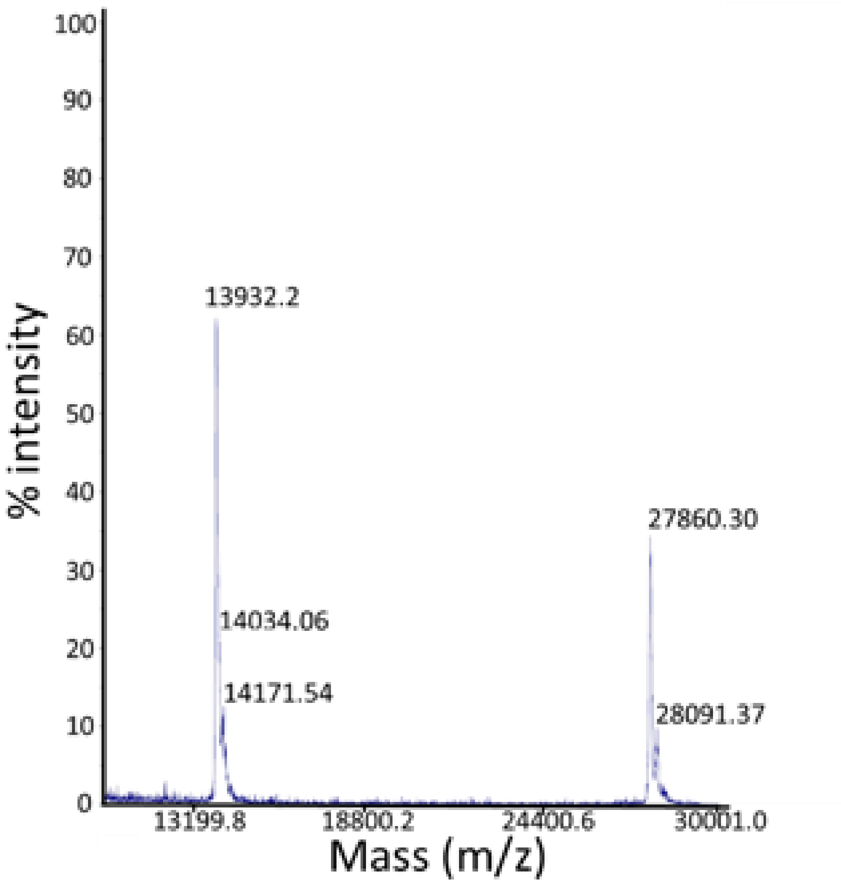

Figure S1. Mass spectrum of cross-linked dimeric TTR obtained using matrix-assisted laser desorption/ionization (MALDI) with time-of-flight (TOF) mass analyzer. Both TTR monomer (13.9 KDa) and dimer (27.9 KDa) were detected in the mass spectrum.

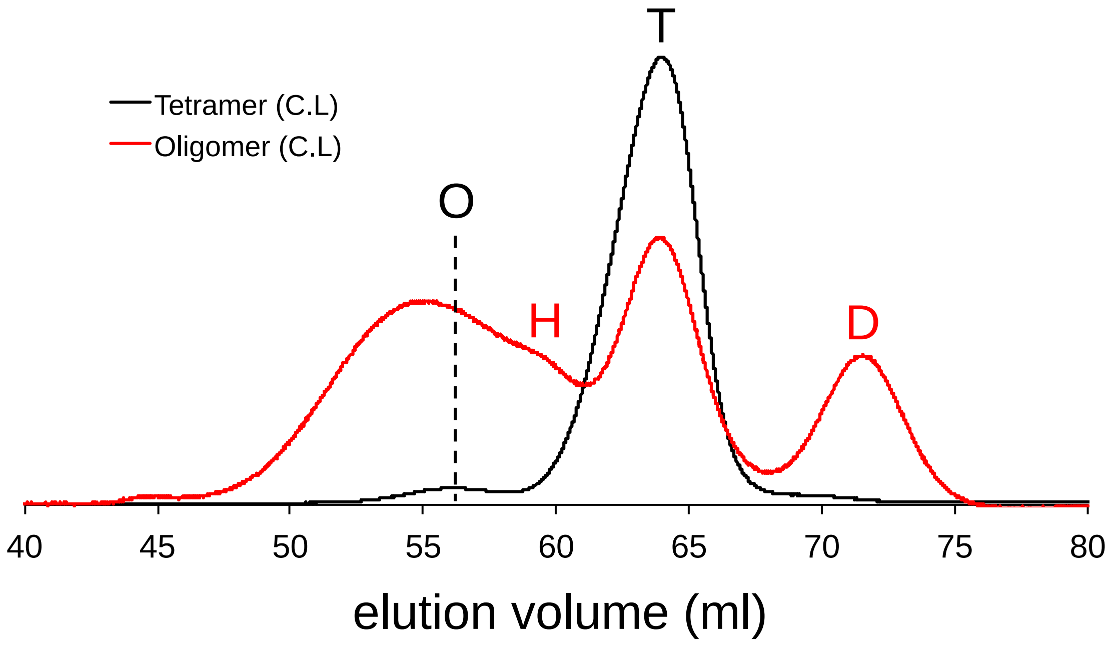

Figure S2. SEC analyses of the cross-linked native tetramers (black) and cross-linked oligomers aggregated from dimers (red). In order to produce TTR octamers, highly concentrated native tetramers (10 mg/ml) were cross-linked and analyzed with SEC (black). The cross-linked tetramers elute at ~ 64 ml, slightly earlier than the uncross-linked tetramers eluting at ~ 66 ml (Figure 1a in the main text), and cross-linked octamers are detected at an elution volume of ~ 56 ml. The cross-linked hexamers elute at ~ 59 ml slightly earlier than intact hexamers eluting at 61 ml in Figure 1b in the main text.

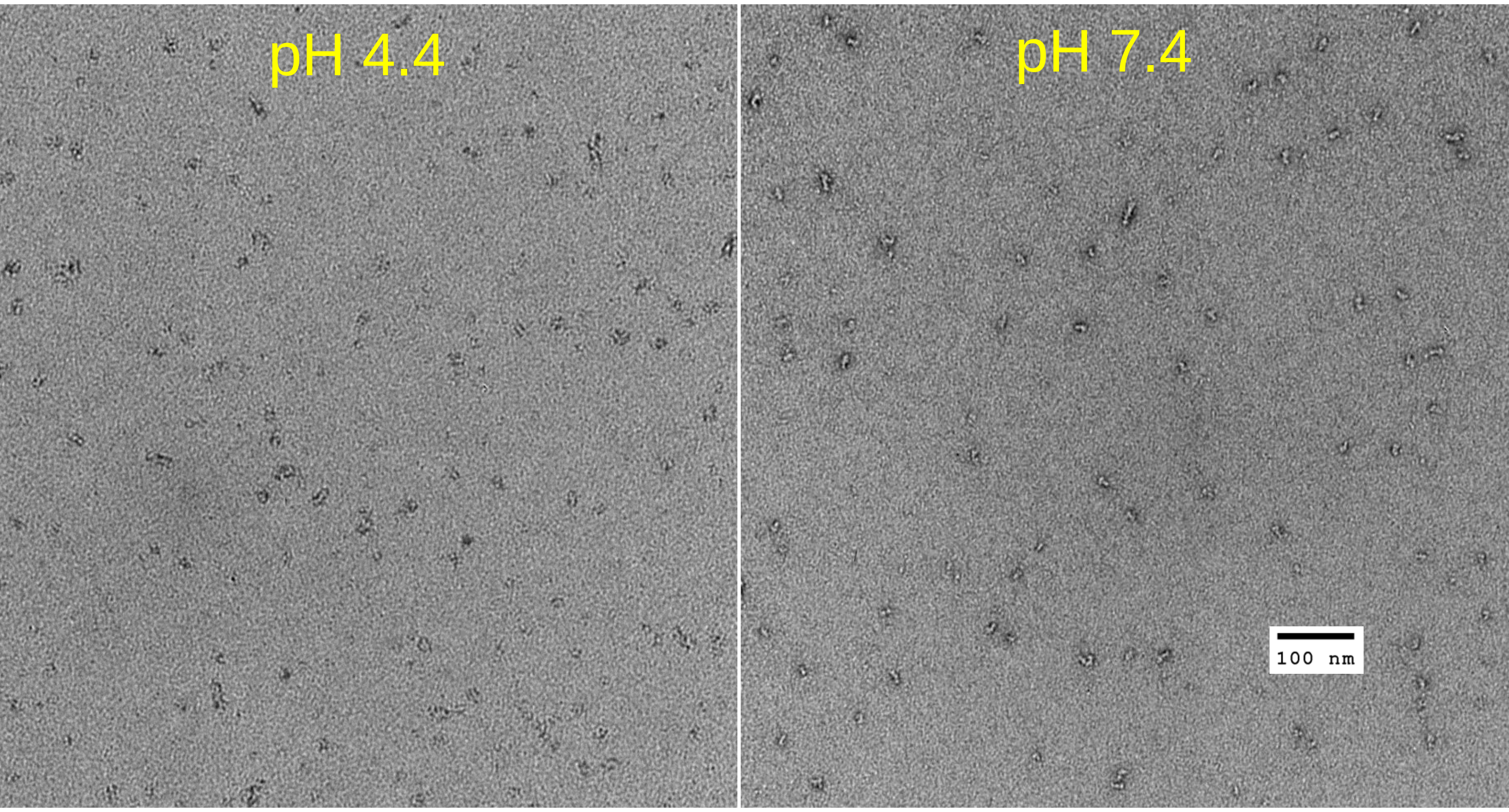

Figure S3. TEM images of the TTR samples (1 mg/ml) incubated for two days at pH 4.4 and 4 °C (left) and after pH was adjusted to 7.4 (right).

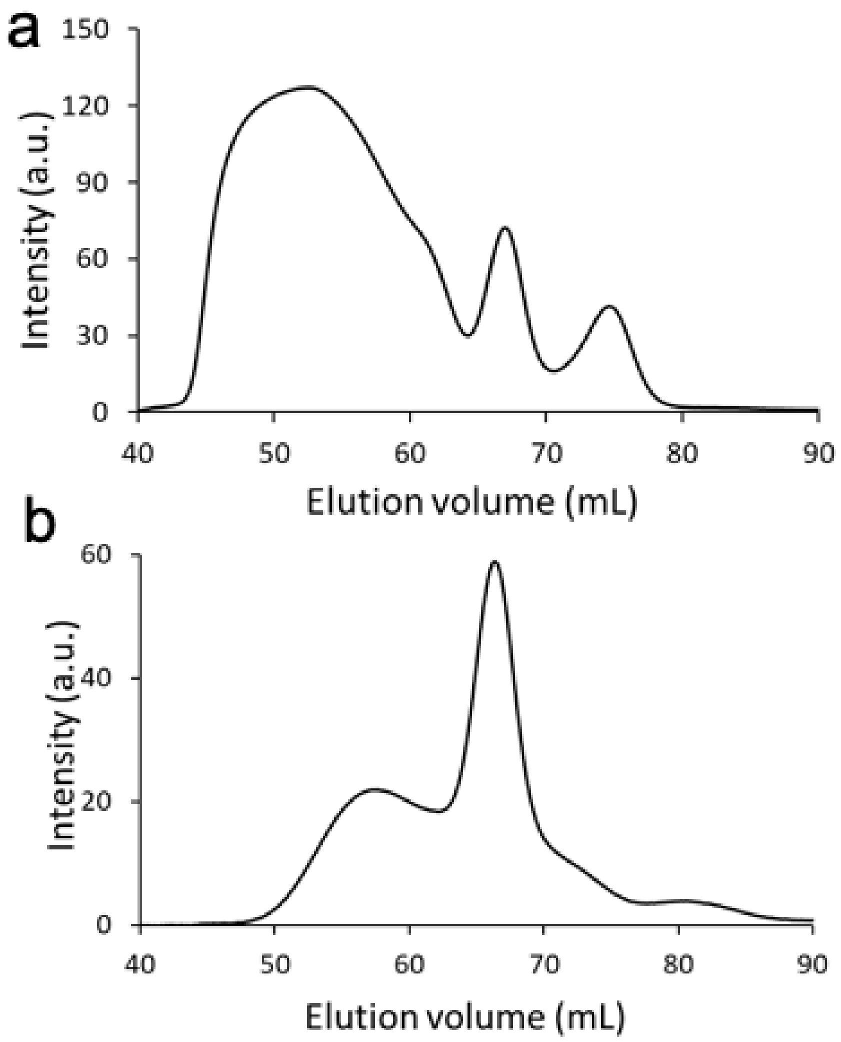

Figure S4. SEC analyses of the WT (a) and G53A (b) oligomers. The WT and G53A oligomers eluting at 55 – 60 ml were collected and used for the cytotoxicity. The two oligomers were prepared as described in the Methods section.

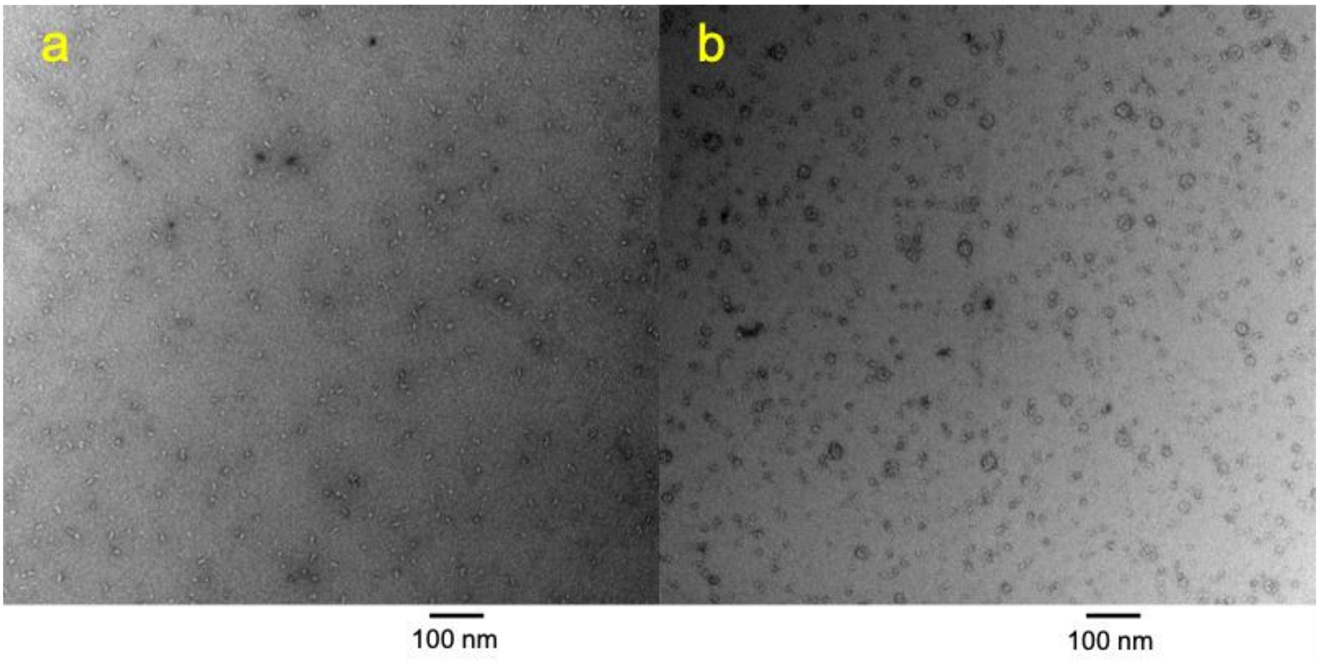

Figure S5. TEM images of the oligomeric species of WT (a) and G53A (b) TTR tested for the cytotoxicity in Figure 3.

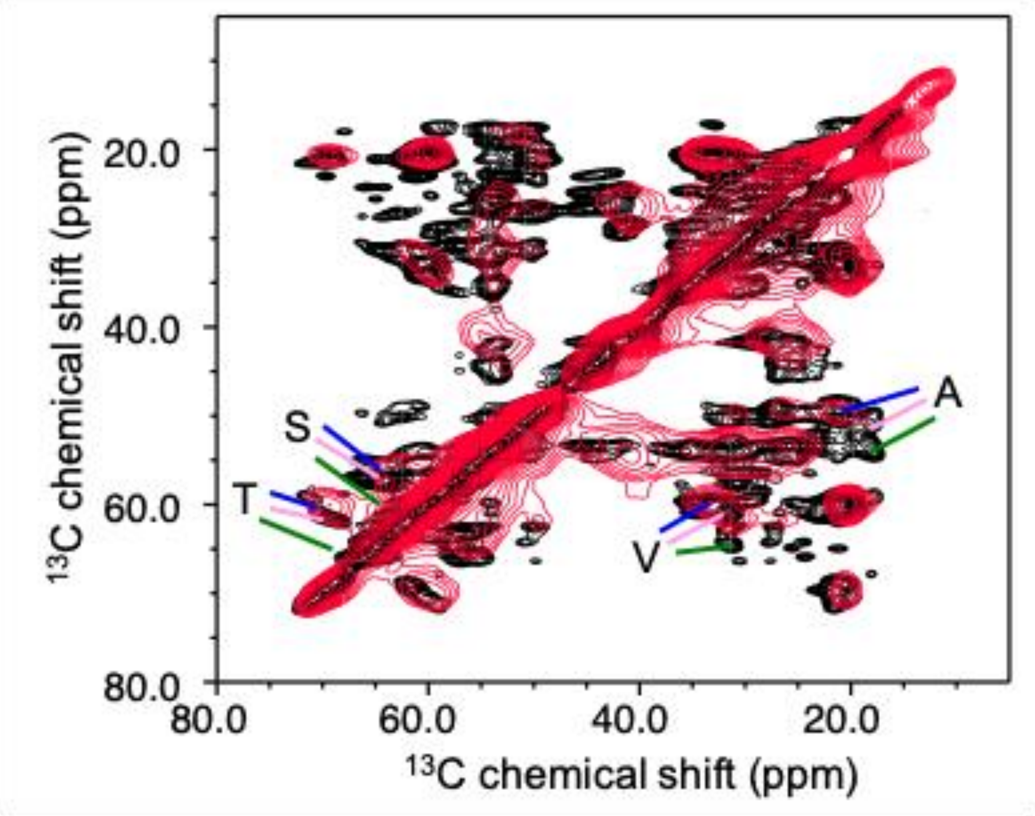

Figure S6. The CP-based two-dimensional ^{13}C-^{13}C correlation solid-state NMR spectra of WT native (black) and WT oligomer (red). The arrows indicate the chemical shift values for the β-sheet (blue), random coil (pink), and α-helical (green) conformation for the four amino acids [1]. The NMR cross-peaks for the Cα and Cβ carbons for the four amino acids in the oligomer spectrum (red) mostly correspond to the β-sheet conformations (blue line).

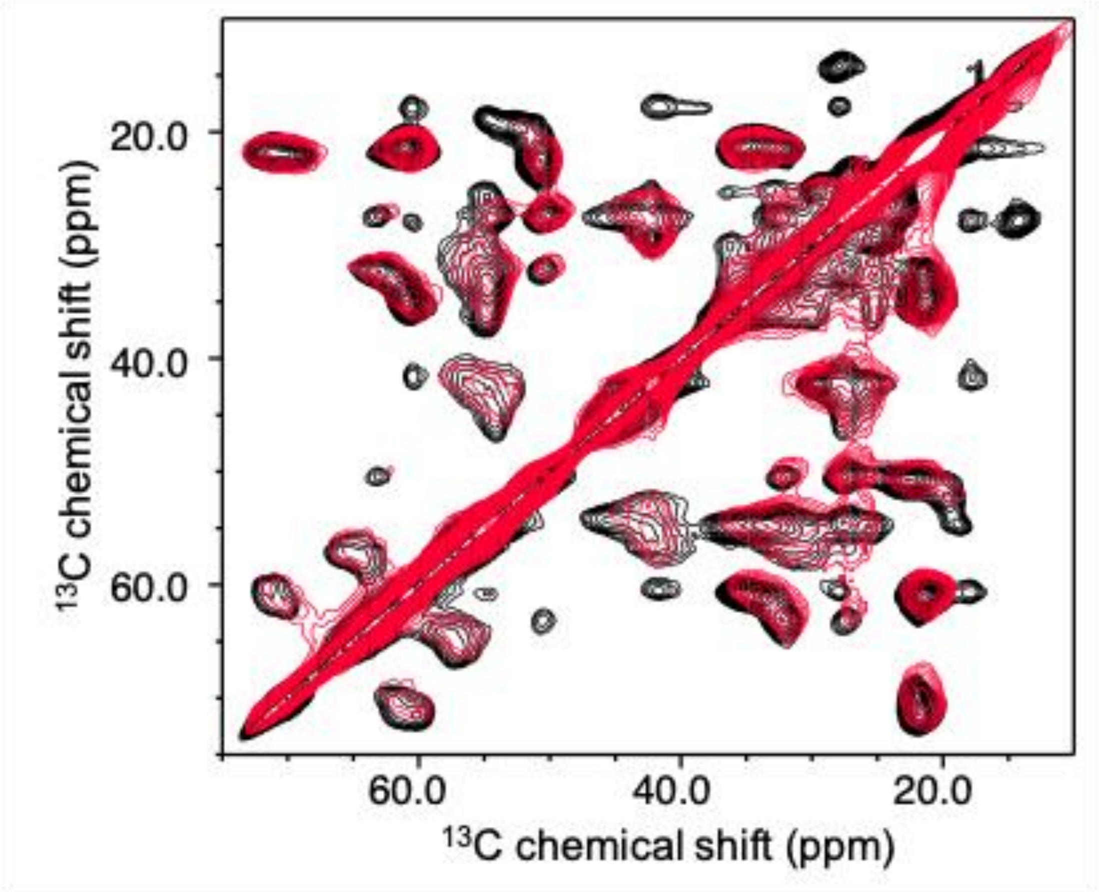

Figure S7. The CP-based two-dimensional ^{13}C-^{13}C correlation solid-state NMR spectra of WT amyloid (black) and WT oligomer (red).

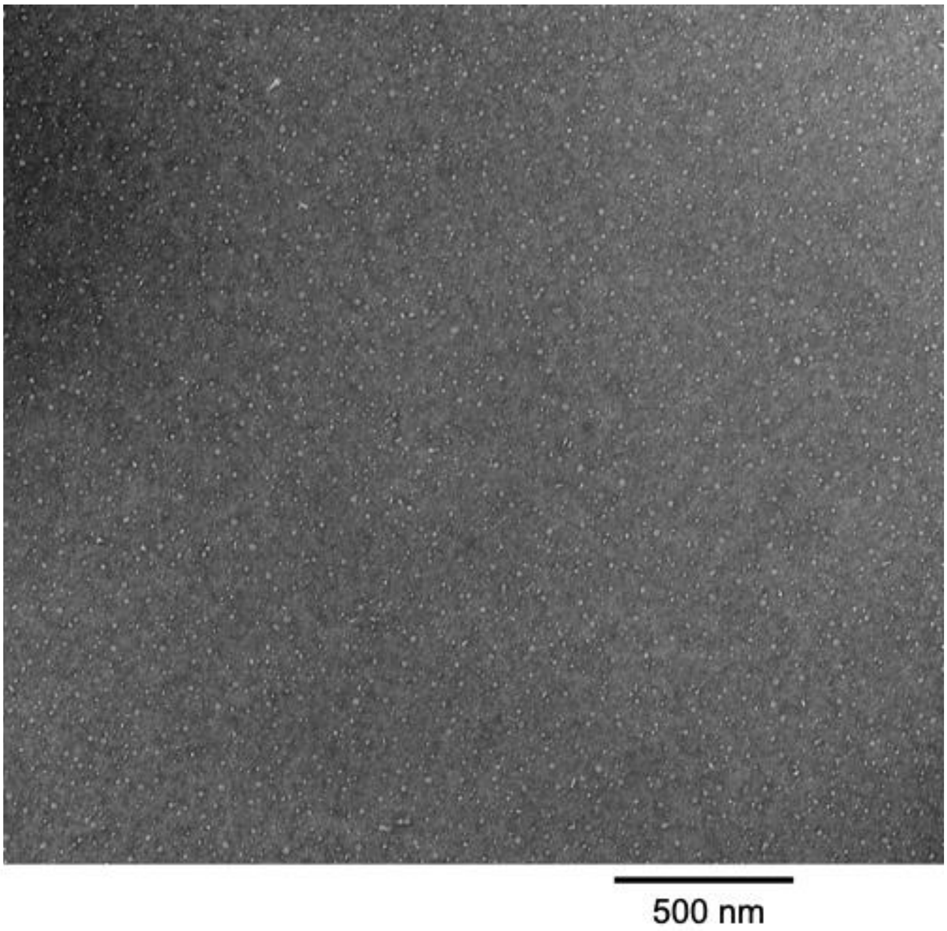

Figure S8. TEM image of the TTR sample in the presence of 10 % ammonium sulfate. WT TTR (0.2 mg/ml) was incubated at pH 4.4 for 7 days and 10 % ammonium sulfate was added to the TTR sample in order to examine the morphology of the oligomers in the presence of the salt.

References

1. Wang, Y. & Jardetzky, O. Probability-based protein secondary structure identification using combined NMR chemical-shift data. *Protein Sci.* **11**, 852-861 (2002).
